# The Impact of COVID-19 Pandemic on Psychopathology and Treatment of People with EDs

**DOI:** 10.1192/j.eurpsy.2022.95

**Published:** 2022-09-01

**Authors:** A.M. Monteleone

**Affiliations:** University of Campania “Luigi Vanvitelli”, Department Of Psychiatry, Naples, Italy

**Keywords:** Psychopathology, Eating Disorders, Treatment, Covid-19 pandemic

## Abstract

Since the beginning of the COVID-19 pandemic a mental health deterioration has been detected in the general population and especially in people affected by pre-existing psychiatric conditions, and mental health care has moved towards online treatment. Literature studies have been reviewed to gather evidence regarding the impact of the COVID-19 pandemic on people affected by eating disorders (EDs) exploring changes in ED-specific and general psychopathology; mechanisms of vulnerability and resilience; change in treatment delivery service; patients’ perception of online treatment and potential barriers and/or advantages of this method and its effectiveness. A wide variability characterized the response of people with EDs to the COVID-19 pandemic. A trend toward worsening of ED specific psychopathology with respect to the pre-pandemic period was observed as well as a deterioration in general psychiatric symptoms. Feelings of uncertainty and social isolation were the most common vulnerability mechanisms. Resilience factors such as heightened self-care and reduced social pressure also occurred. The treatment has largely switched to online delivering strategies which, despite considered the best alternative to the face-to-face approach, were suffering from some concerns about their quality. The exacerbation of specific and general psychopathology supports the hypothesis of an emotional and post-traumatic nature of EDs and outlines the importance of internalizing symptoms. In front of the great variability observed across ED patients during the pandemic, the identification of risk and resilience variables as well as of subjective factors affecting the perception of online treatment may inform clinicians and promote more targeted and individualized approaches.

**Disclosure:**

No significant relationships.

